# How Hybrid Breakdown Can Be Handled in Rice Crossbreeding?

**DOI:** 10.3389/fpls.2020.575412

**Published:** 2020-10-20

**Authors:** Kazuki Matsubara

**Affiliations:** Institute of Crop Science, NARO, Tsukuba, Japan

**Keywords:** genomics-assisted breeding, *Oryza*, reproductive barrier, sterility, weakness

## Abstract

In crosses between genetically divergent parents, traits such as weakness and sterility often segregate in later generations. This hybrid breakdown functions as a reproductive barrier and reduces selection efficiency in crossbreeding. Here, I provide an overview of hybrid breakdown in rice crosses and discuss ways to avoid and mitigate the effects of hybrid breakdown on rice crossbreeding, including genomics-assisted breeding.

## Introduction

Breeders and researchers alike have been interested in hybridization and introgression between divergent genotypes as the potential driver leading to the ecological divergence of progeny (Rieseberg et al., 2007; Arnold et al., 2012). Conversely, hybridization can sometimes involve a reduced hybrid fitness, such as weakness and/or sterility in F_1_ and later generations, even in cases of hybridization between members of the same species.

Reduced hybrid viability and/or fertility segregating in F_2_ or later generations are referred to as hybrid breakdown (HB), in which recessive alleles are necessarily associated. This reproductive barrier has been observed for a long time in both plants and animals (Dobzhansky, 1970; Grant, 1971). Many of the genetic analyses of this barrier have revealed that it is accomplished by a complementary effect between and/or among loci with differentiated alleles, commonly called the Bateson–Dobzhansky–Muller (BDM) incompatibility (Rieseberg and Willis, 2007). HB necessarily involves intrinsic postzygotic reproductive barriers, such as hybrid inviability (including weakness, necrosis, and chlorosis) and hybrid sterility (in the male, female, or both gametes); therefore, some researchers may not distinguish HB from inviability and sterility in the F_1_ progeny. Nevertheless, I believe that HB is a convenient classification for reproductive barriers because it implies their underlying genetic basis (i.e., the involvement of recessive alleles).

Recent studies using the *Arabidopsis* model plant species have provided a better understanding of HB regarding its molecular mechanisms (Vaid and Laitinen, 2019). Conversely, although rice (*Oryza* species) is a model crop, the current understanding of the genetic basis of HB remains limited in this species, probably because HB is not a reproductive barrier in F_1_ hybrids, in which a higher grain production is expected compared with the parental inbred lines, and probably because inferior plants that segregate in F_2_ and later generations can be easily selected out based on the phenotype from the breeding population. The difficulty in genetic mapping caused by recessive inheritance can also be behind this limitation.

Here, I outline HB in rice crosses while referring to information provided by *Arabidopsis* studies, and discuss how HB is handled in rice breeding.

## The Genetic Basis of Hybrid Breakdown in Rice

It is considered that cultivated rice, *O. sativa japonica* and *indica*, forms a species complex with their putative progenitor (*O. rufipogon*) and wild species; however, multiple reproductive barriers, including HB, are observed in crosses among them (Oka, 1988; Vaughan et al., 2003).

To the best of my knowledge, Dr. Oka was the first to describe the genetic basis of HB in intersubspecific crosses between *japonica* and *indica*, which was accounted for by two complementary genes (Oka, 1957). However, the responsible genes were not mapped on chromosomes, because molecular markers were not available at that time.

**FIGURE 1:**
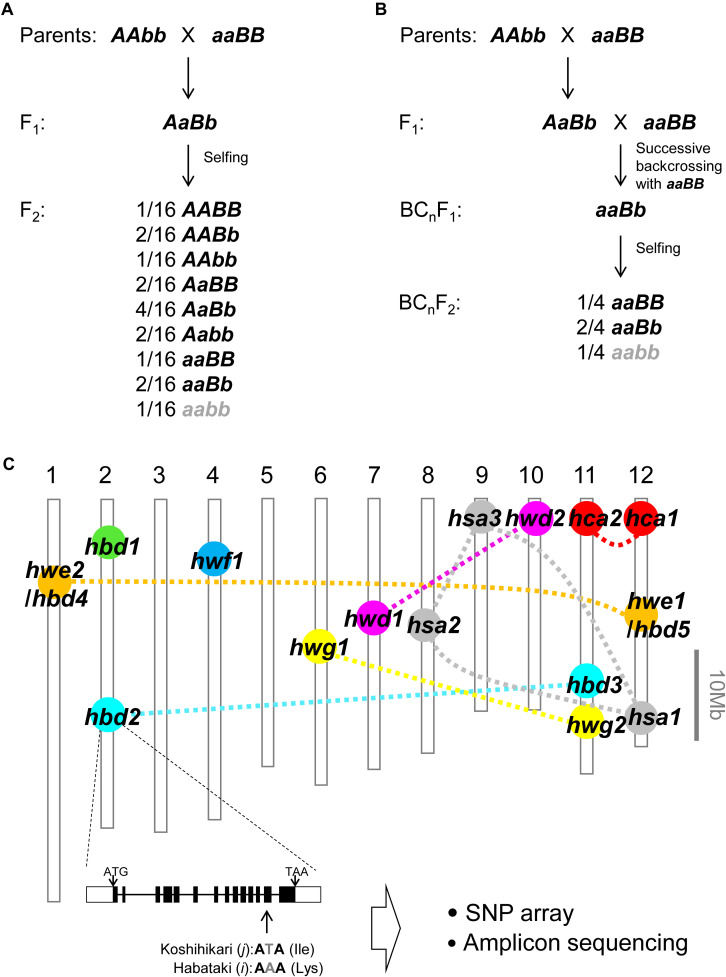
The genetic basis of hybrid breakdown (HB). **(A)** Schematic representation of the HB genotype that segregates in the F_2_ progeny. When the parental genotypes are *AAbb* and *aaBB*, 1/16 of the F_2_ progeny without dominant allele, which is indicated by gray character (i.e., *aabb*), show reduced viability and/or fertility, although the other genotypes are normal. In some rice crosses, the F_2_ progeny with only one dominant allele (i.e., *Aabb*, *aaBb*) also show reduced viability and/or fertility. **(B)** Schematic representation of the HB genotype that segregates in the BC_n_F_2_ progeny. Further, 1/4 of the BC_n_F_2_ progeny shows reduced viability and/or fertility. **(C)** Chromosomal location of genes underlying HB in rice crosses. The genes are roughly mapped based on the results of gene mapping in each study. A set of complementary genes are connected by the dotted line. Complementary genes with *hbd1* and *hwf1* have not been reported. The example of the functional nucleotide polymorphism causing HB published by Yamamoto et al. (2010) is shown, and this information will allow us to conduct a survey by SNP array or amplicon sequencing.

In rice hybrids, HB has often been described in intersubspecific (*O. sativa* ssp. *japonica* × ssp. *indica*) and interspecific (*O. sativa* × *O. nivara* and *O. sativa* × *O. glumaepatula*) crosses (Oka, 1957; Sato and Morishima, 1988; Wu et al., 1995; Li et al., 1997; Fukuoka et al., 1998, 2005; Kubo and Yoshimura, 2002, 2005; Matsubara et al., 2007a,b, 2015; Yamamoto et al., 2007; Ichitani et al., 2012;for intersubspecific crosses; Sobrizal et al., 2001; Miura et al., 2008 for interspecific crosses) (Table 1). Most of these HB cases were caused by two-locus BDM incompatibility and recessive alleles, whereby 1/16 of the F_2_ progeny that was homozygous for recessive alleles at both loci showed the HB phenotype; however, 4/16 of the progeny that was heterozygous at only one locus showed the HB phenotype, depending on the cross combination (Table 1 and Figure 1A). In the backcross hybrids, one locus was already fixed with the alleles from the recurrent parent; therefore, 1/4 of the BC_n_F_2_ progeny exhibited the HB phenotype (Table 1 and Figure 1B).

**TABLE 1:** Genetic basis of the hybrid breakdown reported in rice crosses.

**Cross combination**						**Genotype of weak and/or sterile plant**	**Expected segregation ratio in F_2_ population (Normal, weak, and/or sterile)**	**References**
**Parent 1**			**Parent 2**					
**Variety or accession**	**Species**	**Genotype**	**Variety or accession**	**Species**	**Genotype**			
Sasanishiki	*j*	*Hwd1*/*Hwd1 hwd2*/*hwd2*	Col. No.15	*i*	*hwd1*/*hwd1 Hwd2*/*Hwd2*	*Hwd1*/*hwd1 hwd2*/*hwd2*	11 : 5	Fukuoka et al., 1998
						*hwd1*/*hwd1 Hwd2*/*hwd2*		
						*hwd1*/*hwd1 hwd2*/*hwd2*		
Taichung 65	*j*	*Hwf1*/*Hwf1*	IRGC 105668^a^	*O. glu*	*hwf1*/*hwf1*	*hwf1*/*hwf1*	3 : 1	Sobrizal et al., 2001
Asominori	*j*	*Hwe1*/*Hwe1 hwe2*/*hwe2*	IR24	*i*	*hwe1*/*hwe1 Hwe2*/*Hwe2*	*hwe1*/*hwe1 hwe2*/*hwe2*	15 : 1	Kubo and Yoshimura, 2002
Tachisugata	*j*/*i*	*hbd4*/*hbd4 Hbd5*/*Hbd5*	Hokuriku 193	*i*	*Hbd4*/*Hbd4 hbd5*/*hbd5*	*hbd4*/*hbd4 hbd5*/*hbd5*	15 : 1	Matsubara et al., 2015
Asominori	*j*	*Hsa1*/*Hsa1 hsa2*/*hsa2 Hsa3/Hsa3*	IR24	*i*	*hsa1*/*hsa1 Hsa2*/*Hsa2 hsa3/hsa3*	*hsa1*/*hsa1 hsa2*/*hsa2 hsa3/hsa3*	63 : 1^b^	Kubo and Yoshimura, 2005
Sasanishiki	*j*	*Hwg1*/*Hwg1 hwg2*/*hwg2*	ARC10303	*i*	*Hwg1*/*Hwg1 hwg2*/*hwg2*	*Hwg1*/*hwg1 hwg2*/*hwg2*	11 : 5	Fukuoka et al., 2005
						*hwg1*/*hwg1 Hwg2*/*hwg2*		
						*hwg1*/*hwg1 hwg2*/*hwg2*		
Koshihikari	*j*	*Hbd1*/*Hbd1*	Nona Bokra^a^	*i*	*hbd1*/*hbd1*	*hbd1*/*hbd1*	3 : 1	Matsubara et al., 2007b
Koshihikari	*j*	*Hbd1*/*Hbd1*	IRGC 105444^a^	*O*. *niv*	*hbd1*/*hbd1*	*hbd1*/*hbd1*	3 : 1	Miura et al., 2008
Sasanishiki	*j*	*Hbd2*/*Hbd2 hbd3*/*hbd3*	Habataki	*i*	*hbd2*/*hbd2 Hbd3*/*Hbd3*	*hbd2*/*hbd2 hbd3*/*hbd3*	15 : 1	Matsubara et al., 2007a
Koshihikari	*j*	*Hbd2*/*Hbd2 hbd3*/*hbd3*	Habataki	*i*	*hbd2*/*hbd2 Hbd3*/*Hbd3*	*hbd2*/*hbd2 hbd3*/*hbd3*	15 : 1	Yamamoto et al., 2007
J-147	*j*	*hca1*/*hca1 Hca2*/*Hca2*	IR24	*i*	*Hca1*/*Hca1 hca2*/*hca2*	*hca1*/*hca1 hca2*/*hca2*	15 : 1	Ichitani et al., 2012

**j*, *O. sativa* subspecies *japonica*. *i*, *O. sativa* subspecies *indica*. *j*/*i*, a variety derived from a cross between *j* and *i*. *O. glu*, *O. glumaepatula*. *O. niv*, *O. nivara*. ^*a*^These parents were used as donors to develop chromosomal segment substitution lines of a *japonica* variety (Taichung 65 or Koshihikari). ^*b*^For convenience, semi-sterile segregants were categorized as normal.*

The HB phenotype in rice hybrids varies according to the cross combination. For example, the *hbd2*/*hbd2 hbd3*/*hbd3* genotype shows weakness, but no obvious seed sterility. However, the *hbd4*/*hbd4 hbd5*/*hbd5* genotype exhibits both weakness and seed sterility (Matsubara et al., 2007a, 2015; Yamamoto et al., 2007; Table 1). There are also complicated cases in which HB showing both weakness and seed sterility are observed (*hwe1*/*hwe1 hwe2*/*hwe2* genotype) or HB showing only seed sterility (*hsa1*/*hsa1 hsa2*/*hsa2 hsa3*/*hsa3*) is found, despite the same cross combination, i.e., Asominori × IR24 cross (Kubo and Yoshimura, 2002, 2005; Table 1). As these HBs have independent genetic bases, at least 5/64 of F_2_ progeny would show the HB phenotype in this cross.

To date, several sets of loci responsible for HB have been mapped to particular genomic regions using DNA markers in crosses between the *japonica* and *indica* rice varieties (Chrs 7 and 10, Fukuoka et al., 1998; Chrs 1 and 12, Wu et al., 1995; Kubo and Yoshimura, 2002; Matsubara et al., 2015; Chrs 6 and 11, Fukuoka et al., 2005; Chrs 8, 9, and 12, Kubo and Yoshimura, 2005; Chrs 2 and 11, Matsubara et al., 2007a; Yamamoto et al., 2007, 2010; Chrs 11 and 12, Ichitani et al., 2012; Table 1 and Figure 1C). The results of these studies revealed that loci underlying rice HB are shared in some crosses, but differ in other crosses, which is suggestive of their diversification in rice genomes. For example, in some cases the HB allele carried by *O. nivara*, a species related closely to *incica* varieties, share the same locus with an *indica* variety; however, the HB allele carried by *O. glumaepatula*, a species closely related to cultivated varieties, does not share the locus with any other variety (Table 1 and Figure 1C). It should be noted that the HB cases described above are caused by a set of genes with major effects; however, there are also cases caused by a set of genes with minor effects, e.g., slightly reduced seed fertility, which cannot be overlooked in rice breeding, that have not yet been detected.

## Molecular Mechanisms Underlying Hybrid Breakdown

In *Arabidopsis* hybrids, several molecular mechanisms underlying the BDM type of HB have been experimentally demonstrated, e.g., autoimmune response (Bomblies et al., 2007; Alcázar et al., 2009) and reciprocal silencing of duplicated genes (Bikard et al., 2009; Vlad et al., 2010; Agorio et al., 2017; Blevins et al., 2017).

Autoimmune response: Bomblies et al. (2007) reported first that the autoimmune response that an *NB-LRR* disease-resistance gene or *R* gene is associated with *Arabidopsis* HB in intraspecific crosses, although this autoimmune response is mainly expressed as necrosis in F_1_ plants, and HB in F_2_ progeny seemed to be conditioned by temperature. In *Arabidopsis* hybrids, several lines of evidence of HB caused by the autoimmune response have been described (Alcázar et al., 2009, 2010). The involvement of *NB-LRR* genes in HB suggests that multiple genomic regions can be associated with the *Arabidopsis* HB; in fact, the extensive survey carried out by Chae et al. (2014) supports this idea.

Also in rice hybrids, HB caused by an autoimmune response has been reported in a *japonica* × *indica* cross (Yamamoto et al., 2010). In this case, HB occurs in F_2_ plants when the hybrid breakdown 2 (*hbd2*) gene, which encodes casein kinase I and is carried by the *indica* variety is combined with a cluster of *R* genes carried by the *japonica* variety. As these causative alleles are likely to behave as partially recessive ones, the gene products can be involved (Matsubara et al., 2007a; Yamamoto et al., 2007, 2010).

Reciprocal silencing of duplicated genes: this notion was first theoretically proposed as a genetic basis for hybrid incompatibility (Werth and Windham, 1991; Lynch and Force, 2000). In the model, one population loses function at one locus and retains it at the other, whereas the other population experiences the opposite effect. Consequently, 1/16 of the F_2_ zygotes do not have functional genes (Lynch and Force, 2000). The HB in *Arabidopsis* hybrids described by Bikard et al. (2009) and Vlad et al. (2010) is a good example of this phenomenon. In this scenario, cases involving epigenetic silencing have also been reported (Agorio et al., 2017; Blevins et al., 2017).

As rice has experienced both whole-genome and segmental duplication (Wang et al., 2007; Guo et al., 2019), reciprocal silencing could be prevalent as a cause of hybrid incompatibility. However, to date, there is no evidence of HB caused by reciprocal silencing of duplicated genes in rice.

Independent of the autoimmune response and reciprocal silencing, an interaction between a *DEAD-box RNA helicase 18* and another gene (*MORPHEUS MOLECULE 1* as a likely candidate) that cause HB in *Arabidopsis* hybrids has been identified (Plötner et al., 2017; Vaid et al., 2020). Interestingly, the HB phenotype reduces between the F_3_ and F_4_ generations, implying epigenetic regulation of gene expression.

In rice hybrids, among the three *hybrid sterility-a* (*hsa*) loci, Kubo et al. (2016) recently showed that the *hsa1* locus consists of two genes, and that these genes encode a DUF1618 protein and an uncharacterized protein with some similarity to a nucleotide-binding protein, respectively. The molecular features of the remaining complementary genes, *hsa2* and *hsa3*, have not been reported.

## Hybrid Breakdown in Conventional Rice Breeding

For decades, the bulk-population method was widely employed in conventional rice breeding. In this method, after crossing, the F_4_ or F_5_ population is raised by self-fertilization in bulk (rather than by the single-seed descent method) without artificial selection, but the early generation population is subjected to natural and viability selection (Allard, 1960; Ikehashi and Fujimaki, 1980 for details). In the bulk-population method, many weak and/or sterile genotypes can be expected to be eliminated from the population before the establishment of an advanced-generation population; therefore, this method may provide limited information about HB.

For the introgression or accumulation of desirable traits from donor(s) to a variety, rice breeders have often performed backcrossing or multiple parental crossing, followed by the bulk-population method. Empirically, it has long been known that these crossing methods allow the mitigation of the loss of selection candidates by reproductive barriers, because these methods often reduce the segregation of disruptive combinations of alleles associated with HB in a hybrid progeny. However, the mitigation of reproductive barriers by these crossing methods is inevitably dependent on the HB genotype of the parents.

## Hybrid Breakdown in Genomics-Assited Breeding

The publication of reference crop genome sequences and the development of next-generation sequencing technologies have accelerated the progress of the molecular breeding of crops (Kole et al., 2015). In this context, genomics-assisted breeding, such as genomic selection based on genotypes of genome-wide DNA markers, has been considered in crop breeding (Spindel and Iwata, 2018).

In genomics-assisted breeding of self-pollinated crops, advanced-generation populations, such as recombinant inbred lines, are often used as reference populations, from which genome-wide genotype and phenotype data are obtained. Subsequently, selection based only on marker genotypes is carried out in the progeny of early generations (e.g., F_2_). In this selection scheme, it should be noted that the reference population does not usually provide the information of genomic region for HB, because weak and/or sterile progeny should have been eliminated in the early generations after crossing. Therefore, if such selection scheme is adopted, we will have to abandon some important selection candidates. Alternatively, we may select undesirable candidates such that HB becomes apparent in later generations.

## Discussion

Despite the limited number of studies on this subject, it seems that the loci underlying HB in rice crosses are diversified rather than shared (Table 1 and Figure 1). As described above, the information on the distribution of HB-associated alleles among cross parents should be a prerequisite for rice breeding, particularly for maximizing the effectiveness of genomics-assisted breeding. Therefore, the additional detection of HB in rice crosses and the mapping of responsible genes in the rice genome are needed. In *Arabidopsis* hybrids, such data about the *R* genes and their interacting genes has been extensively surveyed (Alcázar et al., 2010, 2014; Chae et al., 2014). The reporting of the *hbd2* gene by Yamamoto et al. (2010) in a rice hybrid is a good example of an extensive survey performed using SNP arrays or amplicon sequencing (Figure 1C). The *hbd3* and *hsa1* genes are also candidates for this type of survey. Eventually, the development of criteria that allow us to predict HB based on the genomic information of the parental lines will serve as an important tool for genomics-assisted breeding. Even if a causal factor has not been identified as a single gene, closely linked markers, such as single-nucleotide polymorphisms, will be effective for classifying HB-associated haplotypes, because the linkage disequilibrium of cultivated rice has been estimated to extend to 100–200 kb, although that of wild species (such as *O. rufipogon*) may extend over several tens of kb (Mather et al., 2007; McNally et al., 2009; Huang et al., 2010). This information will enable the design of a more efficient and effective cross combination.

Bulked segregant analysis followed by next-generation sequencing can be useful for the mapping of HB-associated loci, as well as conventional linkage mapping (such as quantitative trait locus analysis), because HB segregants often show distinguishable features (about traits such as plant height, tiller number, and fertility) from normal growth segregants in each cross population. The acquisition of imaging data using a digital camera and drone loading may also play an important role in phenotyping in the laboratory and the paddy field. These efforts will provide valuable information not only to rice breeders, but also to evolutionary biologists.

Furthermore, if an HB-associated allele is identified, gene disruption through ion beam or genome editing may help overcome HB, as exemplified by F_1_ hybrid sterility in rice (Koide et al., 2018; Xie et al., 2019).

## Data Availability Statement

All datasets presented in this study are included in the article/supplementary material.

## Author Contributions

The author confirms being the sole contributor of this work and has approved it for publication.

## Conflict of Interest

The authors declare that the research was conducted in the absence of any commercial or financial relationships that could be construed as a potential conflict of interest.
